# Case report of a fatal probable catastrophic antiphospholipid syndrome

**DOI:** 10.3389/fmed.2026.1752865

**Published:** 2026-04-14

**Authors:** Sheng-Jie Hou, Ming-Xin Xie, Xuanlin Feng, Hua Jiang, Ping Zhou

**Affiliations:** 1 School of Medicine, University of Electronic Science and Technology of China, Chengdu, China; 2 Department of Emergency Medicine, Sichuan Provincial People’s Hospital, University of Electronic Science and Technology of China, Chengdu, China

**Keywords:** antiphospholipid antibodies, catastrophic antiphospholipid syndrome (CAPS), infection-triggered CAPS-like episode, splenic infarction, therapeutic plasma exchange (TPE)

## Abstract

Catastrophic antiphospholipid syndrome (CAPS) is an uncommon but often fatal manifestation of antiphospholipid syndrome, marked by rapid, diffuse microvascular thrombosis with multiorgan involvement. In practice, the initial clinical picture can be indistinguishable from severe infection or sepsis-associated organ failure, which makes early diagnosis challenging. Early recognition is critical, as delayed intervention is associated with high mortality. We report a case of probable CAPS that presented with splenic infarction and shock in the setting of suspected sepsis. Her condition progressed quickly to multiorgan dysfunction, and antiphospholipid antibody testing repeatedly showed high-titer triple positivity. After probable CAPS was considered, triple therapy (high-dose glucocorticoids, anticoagulation, and therapeutic plasma exchange) was initiated, and she was discharged after clinical stabilization. She was later readmitted with fulminant infection and recurrent CAPS-like manifestations. Anticoagulation and plasma exchange were not restarted, and she died within 72 h, highlighting two contrasting courses under diagnostic uncertainty.

## Introduction

Catastrophic antiphospholipid syndrome (CAPS) is a fulminant form of antiphospholipid syndrome (APS) with diffuse microvascular thrombosis and rapid multiorgan failure, and reported mortality remains high at about 30%–50% (1). APS is relatively uncommon in the general population, and CAPS represents a very small subset, reported in fewer than 1% of APS cases (1, 2). Early recognition is difficult because CAPS frequently presents with shock and organ dysfunction that closely resemble severe infection and sepsis-associated organ failure (3, 4). Registry and observational studies suggest improved outcomes when anticoagulation, high-dose corticosteroids, and therapeutic plasma exchange are used together. Intravenous immunoglobulin is sometimes used as an adjunct, particularly in severe or refractory cases (3, 5). TPE may help by removing circulating antiphospholipid antibodies and inflammatory mediators, and it is commonly incorporated into combined treatment strategies (3, 6). Here, we report a probable CAPS with initial improvement after combined therapy, followed by a fulminant recurrence during severe infection when anticoagulation and plasma exchange were not restarted.

## Brief medical history

**FIGURE 1:**
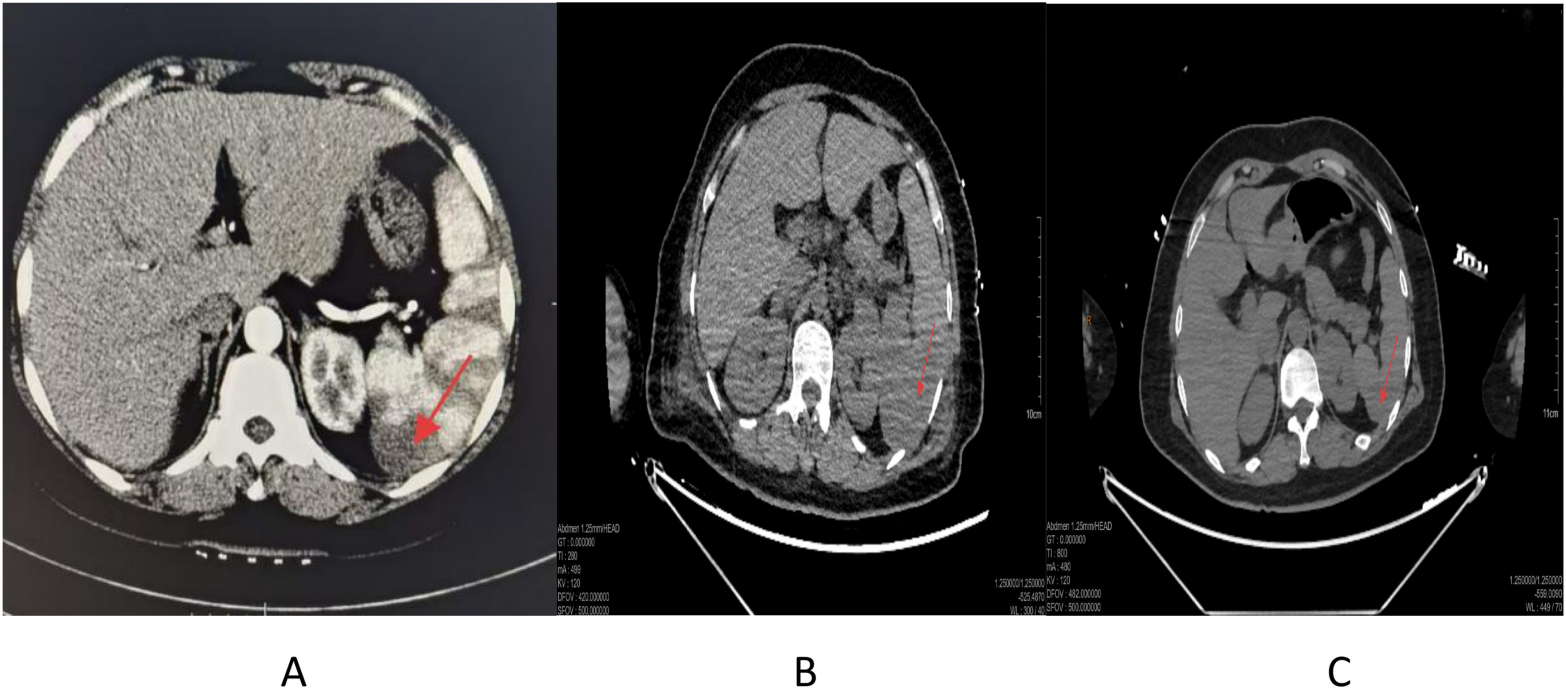
Contrast-enhanced CT images showing the evolution of splenic infarction across two hospitalizations. **(A)** Outside-hospital CT showing multiple wedge-shaped hypodensities in the spleen. **(B)** CT from the initial admission showing multifocal splenic infarcts. **(C)** CT during the recurrent episode showing persistent but slightly reduced infarct areas. Written informed consent for publication of radiologic images was obtained.

The patient, a 42-year-old woman, was admitted with a 10-day history of abdominal pain. At the referring hospital, she had been diagnosed with septic shock due to fever, diarrhea, and persistent hypotension. Contrast-enhanced CT revealed splenic infarction (Figure 1A), while Doppler ultrasonography of the lower limbs showed bilateral deep vein thrombosis. She received endotracheal intubation, broad-spectrum antimicrobial therapy (imipenem plus vancomycin), and vasopressor support; however, her condition did not improve, and she was subsequently transferred to our hospital. On admission, she was confused, with a blood pressure of 95/59 mmHg maintained on norepinephrine at 0.5 μg/kg/min. Her extremities were cold and clammy, and livedo reticularis was evident. The SOFA score was 11, indicating multiorgan dysfunction. Physical examination revealed splenomegaly and symmetrical pitting edema of both lower limbs accompanied by mottled, reticular skin discoloration. Laboratory tests showed a marked inflammatory response, anemia, thrombocytopenia, and acute kidney injury (detailed laboratory values are shown in Table 1). Abdominal CT confirmed multifocal splenic infarction (Figure 1B). Bilateral lower-limb venous thrombosis was confirmed by Doppler ultrasonography, and she had a stillbirth 1 year earlier. Antiphospholipid antibody testing had not been performed at that time. Table 1 summarizes the key laboratory findings, investigations, and treatments over time.

**TABLE 1:** Clinical timeline.

Date/day	Clinical events	Key labs	Imaging	Therapeutic interventions (with dose/volume)	Outcome
Day-10 (outside hospital)	Abdominal pain, diarrhea, fever; MAP 40 mmHg; intubated	WBC 16.24 × 10^9^/L; N 14.73 × 10^9^/L; PCT > 60 ng/mL	Enhanced CT: splenic infarction; lower limb venous color ultrasound: bilateral DVT; CTA: left femoral–GSV stenosis	Imipenem + Vancomycin; vasopressors	No improvement → transferred
Day 0 (admission)	Altered mental status; BP 95/59; mottling; splenomegaly; SOFA 11; APACHE II 22	Hb 57 g/L; PLT 73 × 10^9^/L; Scr 206 μmol/L; CRP 221 mg/L; PCT 10.9; LDH 591; APTT 74.4; D-dimer 2.4	CT: splenic infarction; adrenal enlargement	Ertapenem; fluids; vasopressors	Organ dysfunction worsened
Day 1–3	Fever 39 °C; worsening consciousness; unstable hemodynamics	Hb 64; PLT 53; Scr 322; LDH 631; PCT 3.28; CRP 98.11	—	Supportive therapy	Inflammation ↓ but organ failure ↑
Day 4–7	TMA features; vasopressor-dependent shock; progressive MOF	Coombs (+); aCL IgG 84.7; β2GPI-IgG 133.38; LA screening 206.4; Alternative-etiology workup negative (BM exam; smear/FCM; tumor markers; thrombophilia genetics; ANA/SLE panel).	—	MP 500 mg/day (Day 6–7) IVIG 25 g/day (Day 6–7) LMWH 3000 IU QD (Day 5–6)→Q12 h (Day 7); Piperacillin Sodium and Sulbactam Sodium	MOF progression
Day 8	Critical; renal failure	Scr 350 μmol/L	—	TPE#1 (3,500 mL FFP) MP 500 mg + IVIG 25 g + LMWH 3000 IU q12 h (continued during TPE)	Initial improvement
Day 9	Hemodynamics stabilizing	PLT 68; Scr 314; LDH 249	—	TPE#2 (2,000 mL FFP) MP 160 mg/day LMWH 3000 IU q12 h Discontinue vasopressors	Improving
Day 10	Improved consciousness; defervescence	PLT 77	—	MP taper LMWH 3000 IU q12 h	Stable
Day 11	Clear mentation; stable hemodynamics	PLT 120; Scr 241; LDH 327; aCL 26.06; LA screening 81.2	—	TPE#3 (2,200 mL FFP) MP 40 mg bid LMWH 3000 IU q12h	Marked improvement
Day 12	Stable	PLT 125; Scr 197; LDH 292	—	TPE#4 (3,000 mL FFP) MP taper LMWH 3000 IU q12 h	ICU step-down
Day 13–14	Stable on ward	aCL 22.76; β2GPI-IgG 34.9; LA screening 63.1	—	LMWH 6000 IU QD + Pred 50 mg	Ward transfer
Day 15–23	Clinically stable	aCL 39.78; β2GPI-IgG 66.51	—	LMWH Day 15–17→Warfarin 2.5 mg/day + Pred	Stable
Day 24 (discharge)	Stable	—	—	Pred 20 mg/day; Warfarin 3.5 mg/day	Discharged
2 months post-discharge	Stable outpatient follow-up	aCL 62.34; LA screening 83.7; β2GPI-IgG 82.65	—	Continued anticoagulation + low-dose steroids	Persistent aPL
Re-admission Day 0	Fever 39 °C; shock; mottling; altered mentation; sepsis + MOF	CRP 180 mg/L; PCT 160; IL-6 8341; PLT 71; Scr 335; INR 7.58; D-dimer 0.32	CT: splenic infarction; new hyperdensity in the right adrenal gland	Intubation; NE + terlipressin; MP 80 mg q8 h; IVIG 20 g; Meropenem	Severe deterioration
Re-admission Day 1–2	Rapid deterioration; progressive MOF	LDH 1235; TnT 945; NT-proBNP > 70,000	—	No anticoagulation; no TPE	Cardiorenal failure
Re-admission Day 3	Malignant arrhythmia; circulatory collapse	aCL IgG 82.07; β2GPI-IgG 138.88; LA screening 220.8	—	Multiple defibrillation attempts	Death

WBC, white blood cell count; N, neutrophil count; Hb, hemoglobin; PLT, platelet count; CRP, C-reactive protein; PCT, procalcitonin; IL-6, interleukin-6; Scr, serum creatinine; TB, total bilirubin; DB, direct bilirubin; LDH, lactate dehydrogenase; APTT, activated partial thromboplastin time; PT, prothrombin time; INR, international normalized ratio; DVT, deep vein thrombosis; CTA, computed tomography angiography; GSV, great saphenous vein; TMA, thrombotic microangiopathy; MOF, multiorgan failure; MP, methylprednisolone; IVIG, intravenous immunoglobulin; LMWH, low-molecular-weight heparin; TPE, therapeutic plasma exchange; FFP, fresh frozen plasma; NE, norepinephrine; anticardiolipin antibodies (aCL), lupus anticoagulant (LA) screening, and anti-β2-glycoprotein I (β2GPI) antibodies; BM, bone marrow; FCM, flow cytometry; ANA, antinuclear antibody; SLE, systemic lupus erythematosus. Units and reference ranges: WBC, N: × 10^9^/L (Ref: 3.5–9.5); Hb: g/L (Ref: 115–150); PLT: × 10^9^/L (Ref: 125–350); CRP: mg/L (Ref: <5); PCT: ng/mL (Ref: <0.05); IL-6: pg/mL (no fixed reference; extreme elevation clinically significant); Scr: μmol/L (Ref: 45–90 for females); TB: μmol/L (Ref: 5–21); DB: μmol/L (Ref: 0–7); LDH: U/L (Ref: 135–225); APTT: seconds (Ref: 25–35); PT: seconds (Ref: 11–15); D-dimer: mg/L FEU (Ref: < 0.5). aCL IgG: U/mL (Ref:0–20); β2GPI-IgG: U/mL (0–20); LA screening: s (Ref:31–44); “—” indicates that the test was not performed or the value was unavailable.

From Day 1 to Day 3, the patient remained febrile with persistent altered consciousness and ongoing hemodynamic instability despite supportive care and antimicrobial therapy (Table 1 and Supplementary Table 3). During the initial admission, we performed repeated microbiological testing, including bronchoalveolar lavage culture, blood and urine cultures, stool culture, blood metagenomic sequencing, and respiratory viral testing, but none identified a causative pathogen (Supplementary Table 3). From Day 4 onward, the alternative workup was unrevealing, and anemia and thrombocytopenia progressed with rising LDH (Table 1). Fresh frozen plasma and platelet transfusions were given during this period (Table 1). Protein C activity was 47%, protein S activity 123%, and ADAMTS13 activity 105% (Supplementary Figure 4), while aPL testing showed high-titer triple positivity (Table 1). Detailed aPL testing is provided in Supplementary Table 1.

**FIGURE 2:**
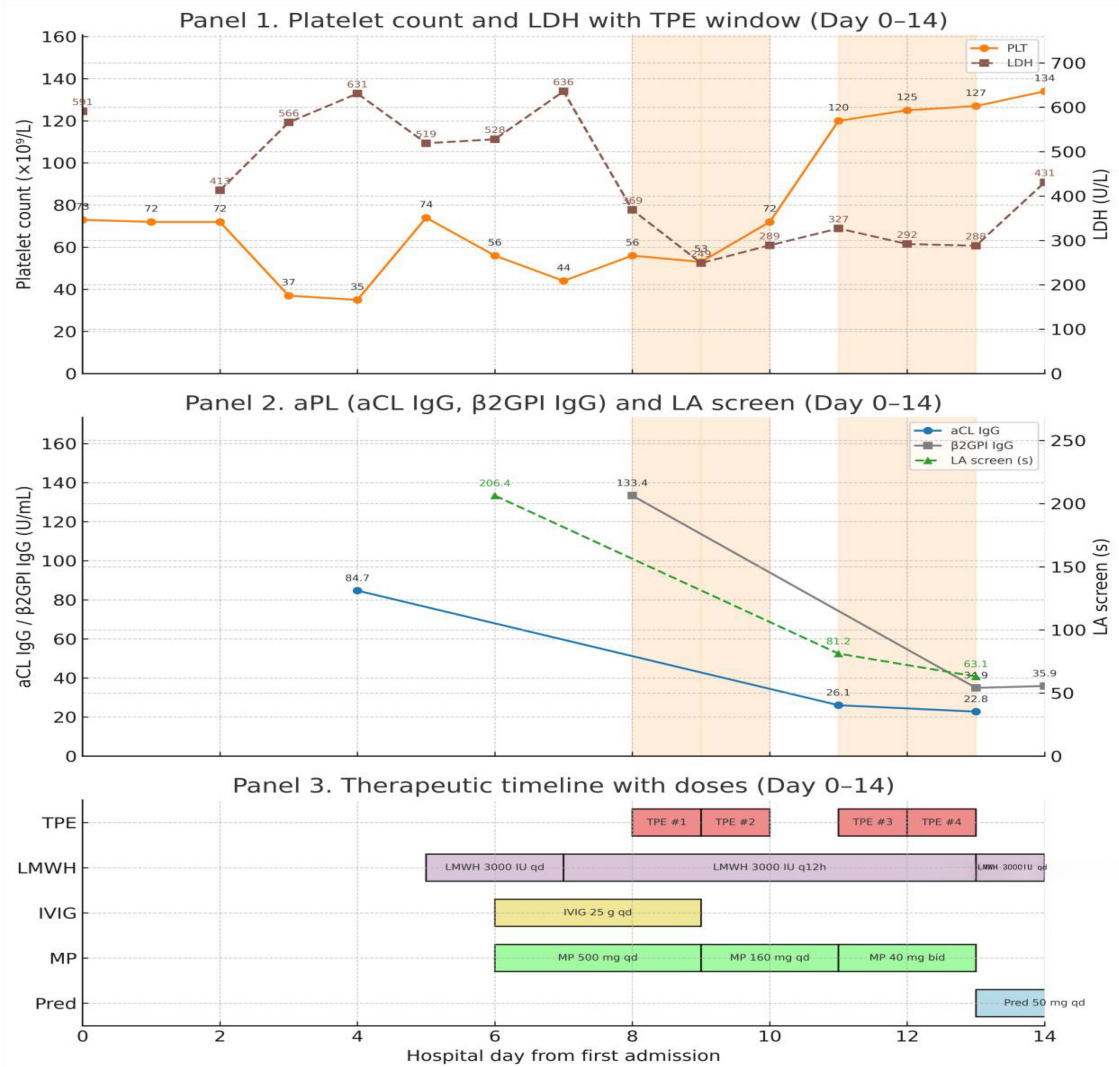
Laboratory trajectories and treatment timeline during the initial probable catastrophic antiphospholipid syndrome (CAPS) episode.

By Day 7, despite supportive care and antimicrobial therapy, the patient exhibited persistent shock, worsening renal function, and progressive thrombocytopenia. Because her clinical course progressed rapidly, with thrombotic microangiopathy features and high-titer antiphospholipid antibodies, we initiated treatment for suspected CAPS. Therapeutic anticoagulation and high-dose glucocorticoids were started, with IVIG as adjunctive treatment. Anticoagulation was maintained during the plasma exchange sessions (Table 1 and Figure 2). As clinical instability persisted, plasma exchange was added to complete triple therapy. Hemodynamics then stabilized and vasopressors were discontinued. Temperature normalized and mental status recovered. Laboratory values improved, and she was successfully extubated (Table 1). After 13 days in the ICU, she was transferred to the general ward, where her platelet count continued to rise, LDH approached normal levels, and aPL titers continued to decline. She was discharged in stable condition on day 24 with oral prednisone and warfarin.

At the 2-month follow-up, aPL titers remained positive, and she continued anticoagulation with low-dose glucocorticoids. Four months later, glucocorticoids were discontinued as advised. Three months later, the patient was readmitted with high fever, shock, livedo reticularis, and impaired consciousness. On readmission, laboratory tests showed markedly elevated inflammatory markers (CRP, procalcitonin, and interleukin-6), thrombocytopenia, and worsening renal function, with concurrent deterioration of cardiac and hepatic function; antiphospholipid antibody titers were again strongly positive (Table 1). Imaging demonstrated persistent splenic infarction (Figure 1C) with new hyperdensity in the right adrenal gland (Supplementary Figure 5). During this admission, she was treated with broad-spectrum antibiotics, vasopressors, methylprednisolone, and IVIG. Anticoagulation was not restarted because PT and INR were markedly prolonged. Plasma transfusion was administered for coagulopathy, but PT/INR remained markedly prolonged throughout the short clinical course (Supplementary Table 2). Plasma exchange was not initiated because sepsis dominated the presentation and aPL results were not yet available. Her condition deteriorated rapidly, and she died within 72 h due to malignant arrhythmia.

The figure illustrates laboratory trajectories in relation to the stepwise initiation of combined therapy. Red shaded areas denote the timing of plasma exchange sessions. Declines in antiphospholipid antibody titers and improvement in laboratory parameters were observed following initiation of triple therapy. Several treatments were introduced in close succession, so the figure shows timing rather than treatment attribution (5, 7, 8).

## Discussion

Catastrophic antiphospholipid syndrome should be considered early when shock, multiorgan dysfunction, new thrombosis, and strongly positive aPL coexist, even while sepsis is being treated (3, 9). The main difficulty in this case was that severe infection and CAPS shared the same clinical picture, so it was hard to determine which process was driving deterioration at the bedside. The first admission fulfilled the modified Asherson criteria for probable CAPS (Table 2) (4). In the second admission, fulminant infection dominated the presentation, which further reduced the diagnostic window for initiating combined therapy in a CAPS-like episode. The contrasting courses across the two admissions show how hard it can be to make early diagnostic and treatment decisions in this setting.

**TABLE 2:** Application of catastrophic antiphospholipid syndrome (CAPS) classification criteria to the present case.

CAPS classification criterion	Findings in this case	Fulfilled?
≥3 organ systems involved	Spleen infarction, bilateral DVT, livedo reticularis/skin mottling	Yes
Rapid progression (1 week)	Multiorgan failure evolved within 72 h	Yes
Histologic evidence of small-vessel thrombosis	No biopsy available; TMA features indirect (PLT↓, LDH↑, anemia, Coombs+)	Partial
aPL positivity (≥1 test at high titer)	High-titer aCL, β2GPI, and LA at admission and persistent positivity at 2 months	Yes

According to the International Consensus Criteria (Asherson et al., Lupus), definite CAPS requires involvement of ≥3 organs, rapid onset (1 week), histopathological confirmation of small-vessel thrombosis, and laboratory confirmation of aPL. Probable CAPS may be diagnosed when criteria 1, 2, and 4 are fulfilled in the absence of histopathological confirmation, or under other predefined modified combinations (4).

At presentation, the patient met the clinical criteria for septic shock, and broad-spectrum antimicrobial therapy was initiated on admission. Inflammatory markers and leukocyte counts decreased during the first few days. However, repeated microbiological investigations within the first week remained negative, and no definite infectious focus was identified (Supplementary Table 3). Despite this, organ dysfunction continued to worsen, with progressive renal impairment, worsening thrombocytopenia, and persistent hemodynamic instability. Norepinephrine was required at 0.25–0.5 μg/kg/min (Table 1 and Supplementary Table 2). Sepsis was treated, but deterioration persisted, suggesting additional processes contributed. The patient developed multisystem thrombosis within a short period and showed persistently high-titer triple-positive antiphospholipid antibodies (Table 1). Tissue biopsy was not available, so we could not confirm microvascular thrombosis histologically; however, the clinical course and antibody profile supported probable CAPS (4).

Sepsis can trigger CAPS and overlaps with its clinical manifestations (3, 10). Negative microbiology does not exclude sepsis, particularly after prior antibiotics. Accordingly, the dominant driver early in the course could not be determined. Infection can be associated with transient aPL positivity, which is typically isolated rather than triple-positive (10, 11). An intensive care unit cohort study reported lupus anticoagulant positivity in 52.9 percent of patients, without detectable anticardiolipin or anti-β2GPI antibodies (12). In this patient, high-titer triple positivity persisted during follow-up, which is difficult to attribute to infection alone (Table 1). Her coagulation profile did not fit a typical pattern of sepsis-associated consumptive coagulopathy or disseminated intravascular coagulation. There was no sustained fibrinogen depletion and no marked elevation in D-dimer levels (Supplementary Table 2) (13–15). ADAMTS13 activity was normal, making classical immune-mediated thrombotic thrombocytopenic purpura unlikely (16).

With high-titer aPL and progressive multiorgan dysfunction, we initiated triple therapy, and IVIG was given as adjunctive immunomodulatory therapy (Table 1) (3, 5). The 2023 American Society for Apheresis (ASFA) guidelines classify CAPS as a Category I indication for the use of TPE, recommending daily or alternate-day exchange of 1–1.5 plasma volumes with FFP as the replacement fluid (17). In this patient, four sessions of TPE were performed, with individual exchange volumes ranging from 2000 to 3500 mL. The estimated exchange volume was approximately 1.2 times the patient’s plasma volume, in accordance with guideline recommendations. FFP was used throughout, and no adverse reactions occurred. After initiation of combined therapy, the clinical course stabilized (Figure 2, Table 1, and Supplementary Table 2). The rapid turnaround mirrors reports of triple therapy in CAPS, although several interventions were started in parallel, precluding attribution to any single component (5, 7, 9, 18, 19).

In follow-up, low-dose glucocorticoids were discontinued as advised. Long-term glucocorticoids are not standard therapy for APS (20); in CAPS, they are used acutely as part of combined treatment. At the second admission, fulminant infection coincided with high-titer aPL and rapid multiorgan involvement (Table 1); we therefore describe it as a CAPS-like episode, given the absence of histopathology and major diagnostic overlap with sepsis (Tables 1, 3) (3, 4, 9). The adrenal imaging abnormality may also suggest additional organ involvement during the recurrent episode. In addition, the patient showed a markedly elevated INR without a corresponding rise in D-dimer (Supplementary Table 2). Given that her INR during outpatient warfarin follow-up had remained therapeutic (around 2.0–2.5), the abrupt prolongation was likely related to warfarin over-anticoagulation in the setting of acute illness. This pattern is not typical of DIC (13, 15), where D-dimer is usually markedly elevated, and it is more consistent with warfarin over-anticoagulation during acute illness.

**TABLE 3:** Key differences between the initial episode and the subsequent fulminant episode with catastrophic antiphospholipid syndrome (CAPS)-like features.

Domain	Initial episode	Recurrent episode	Interpretation
Trigger	Infection-associated episode	Severe infection with APS background	High-risk setting with infection as the dominant trigger.
aPL profile	High-titer aPL	Persistently high-titer aPL	Persistent thrombotic potential
Organ involvement	Spleen, bilateral DVT, livedo reticularis/skin mottling	Spleen progression, adrenal lesion, skin, severe MOF	Multi-organ involvement in both episodes
Inflammation	Moderate inflammatory response	Fulminant cytokine surge	Infection-triggered flare
Hemodynamics	Shock responsive to therapy	Refractory shock	Uncontrolled microvascular thrombosis
Renal function	Reversible AKI	Rapidly worsening AKI	Loss of renal recoverability
TMA markers	Moderate TMA features	Severe TMA features	Aggressive endothelial injury
Treatment	triple therapy (anticoagulation + corticosteroids + plasma exchange), with adjunctive IVIG	Suboptimal therapy (no TPE; no anticoagulation)	Delayed or incomplete implementation of triple therapy
Outcome	Full recovery	Rapid fatal deterioration	Outcomes differed across episodes in the setting of distinct clinical contexts and treatment implementation

This table summarizes major clinical, laboratory, and treatment contrasts between the initial episode and the subsequent fulminant episode.

We also noted that this patient had shown rapid improvement during the initial episode when treated with combined high-dose glucocorticoids, anticoagulation, IVIG, and TPE. However, during the subsequent fulminant episode, early anticoagulation and TPE were not initiated because priority was given to treating sepsis and concerns over bleeding risk. In the CAPS Registry analysis, mortality was 28.6% with triple therapy, 41.1% with other combinations of these drugs, and 75% when none of the triple therapies were used (5). In this case, the incomplete implementation of triple therapy during the subsequent episode may have been associated with the different outcome, but this does not establish causality. Key contrasts are summarized in Table 3. Complement inhibition (e.g., eculizumab) has been reported as rescue therapy in refractory CAPS; however, it was not used in this case, and complement tests were not measured during either admission (21–23).

## Conclusion

In patients with sepsis-like shock plus thrombotic features, our case supports considering CAPS early and discussing combined therapy when suspicion is high. Infection is a common trigger of CAPS and can obscure early recognition, particularly in patients with rapid thrombosis and persistently high-risk aPL profiles (3, 10).

## Data Availability

The original contributions presented in this study are included in this article/Supplementary materials, further inquiries can be directed to the corresponding authors.
