# Supplementary material for: Case report of a fatal probable catastrophic antiphospholipid syndrome
Source: Front Med (Lausanne). 2026 Apr 14;13:1752865. doi: 10.3389/fmed.2026.1752865 (PMC13121309; doi:10.3389/fmed.2026.1752865)
Supplement: Supplementary file 1 [file Table_1.docx]

# Supplementary Table S1. Longitudinal antiphospholipid antibody (aPL) profile

| **Day** | **aCL IgM**  **(U/mL)** | **aCL IgG**  **(U/mL)** | **aCL IgA**  **(U/mL)** | **LA ratio** | **LA screen**  **(sec)** | **LA confirm**  **(sec)** | **β2GPI IgM**  **(U/mL)** | **β2GPI IgG**  **(U/mL)** | **β2GPI IgA**  **(U/mL)** |
| --- | --- | --- | --- | --- | --- | --- | --- | --- | --- |
| Day 4 | 14.45 | 84.72 | 14.85 | — | — | — | — | — | — |
| Day 8 | — | — | — | — | — | — | 3.69 | 133.38 | 14.69 |
| Day 11 | 39.66 | 26.06 | 10.05 | 2.56 | 81.2 | 31.7 | — | — | — |
| Day 13 | 22.92 | 22.76 | 6.05 | 1.95 | 63.1 | 32.4 | 22.8 | 34.94 | 3.27 |
| Day 14 | — | — | — | — | — | — | 30.05 | 35.89 | 4 |
| Day 18 | 29.44 | 39.78 | 11.12 | — | — | — | 29.1 | 66.51 | 5.88 |
| Two months post-discharge | 12.43 | 62.34 | 45.59 | 2.14 | 83.7 | 39.1 | 4.22 | 82.65 | 16.26 |
| Re-admission Day 3 | 25.93 | 82.07 | 26.38 | 3.64 | 220.8 | 59.1 | 2 | 138.88 | 12.51 |

**Notes:**
This table summarises the temporal profile of antiphospholipid antibodies (aPL) during the patient's initial CAPS-like episode, follow-up period, and recurrent presentation. The measurements include anticardiolipin antibodies (aCL), lupus anticoagulant (LA) parameters, and anti-β2-glycoprotein I (β2GPI) antibodies. “—” indicates that the test was not performed or the value was unavailable.

Reference ranges:aCL IgM / IgG / IgA: 0–20 U/mL;β2GPI IgM / IgG / IgA: 0–20 U/mL;LA ratio: 0.8–1.2;LA screening: 31–44 s;LA confirmation: 33–38 s.
