# Supplementary material for: Case report of a fatal probable catastrophic antiphospholipid syndrome
Source: Front Med (Lausanne). 2026 Apr 14;13:1752865. doi: 10.3389/fmed.2026.1752865 (PMC13121309; doi:10.3389/fmed.2026.1752865)
Supplement: Supplementary file 2 [file Table_2.docx]

# **Supplementary Table S2. Laboratory Parameters During Hospitalisation**

| **Day** | **WBC** | **Hb** | **PLT** | **CRP** | **PCT** | **Cr** | **TB** | **DB** | **LDH** | **APTT** | **PT** | **D-Dimer** | **FIB** |
| --- | --- | --- | --- | --- | --- | --- | --- | --- | --- | --- | --- | --- | --- |
| Day 0 | 7.74 | 57 | 73 | 221.16 | 10.9 | 206 | 23.4 | — | 591 | 74.4 | 15.7 | 2.94 | 8.16 |
| Day 1 | 11.53 | 68 | 72 | 203 | 10.5 | 209.4 | 19.3 | 6.5 | — | 58.9 | 15.8 | 2.08 | 8.02 |
| Day 2 | 5.1 | 77 | 72 | 105.51 | 6.92 | 233.3 | 14.8 | 9 | 413 | 51.8 | 12.8 | 4.24 | 6.64 |
| Day 3 | 4.9 | 85 | 37 | 98.11 | 3.28 | 262.3 | 19.7 | 10 | 566 | 69.5 | 14.2 | 17.43 | 11.51 |
| Day 4 | 6.02 | 86 | 35 | 138.88 | — | 276.8 | 18.7 | 12.7 | 631 | — | — | — | — |
| Day 5 | 8.35 | 79 | 74 | 129.63 | 2.4 | 303.2 | 15.5 | 10.2 | 519 | 67.6 | 15.7 | 14.56 | 8.93 |
| Day 6 | 6.22 | 82 | 56 | 174.25 | 1.96 | 322.4 | 15.2 | 9 | 528 | 74.5 | 13.7 | 9.98 | 9.66 |
| Day 7 | 8.15 | 71 | 44 | 163.49 | 1.34 | 217.3 | 9.8 | 4.4 | 636 | 66 | 12.5 | 4.37 | 8.99 |
| Day 8 | 17.1 | 64 | 56 | 27.41 | — | 350 | 9.0 | 5.1 | 369 | 38.4 | 12 | 3.09 | 3.29 |
| Day 9 | 10.6 | 68 | 53 | 8 | 0.63 | 314 | 11.3 | 9.6 | 249 | 35.4 | 11.6 | 3.17 | 1.88 |
| Day 10 | 10.42 | 77 | 72 | 4.83 | 0.34 | 282 | 15.6 | 8.1 | 289 | 38.5 | 11.8 | 3.78 | 1.7 |
| Day 11 | 7.39 | 77 | 120 | 5.07 | 0.34 | 241.3 | 15.9 | 10.1 | 327 | 34.7 | 11.3 | 9.38 | 1.77 |
| Day 12 | 8.92 | 83 | 125 | 4.56 | 0.31 | 197 | 16.6 | 4.8 | 292 | 31.5 | 11.6 | 7.2 | 1.83 |
| Day 13 | 8.97 | 74 | 127 | 3.07 | 0.25 | 174 | 12.9 | 8.7 | 288 | 36.8 | 11.3 | 7.38 | 1.75 |
| Day 14 | 7.49 | 78 | 134 | 2.45 | 0.17 | 154.3 | 14.4 | 10.6 | 431 | 46.6 | 11 | 4.63 | 2.18 |
| Day 16 | — | — | — | — | — | — | — | — | — | 46.7 | 13.7 | 7.12 | 3.2 |
| Day 17 | 13.34 | 100 | 203 | 2.66 | — | 224 | 14.1 | 5.6 | 340 | 49.7 | 14.9 | 7.54 | 3.58 |
| Day 19 | — | — | — | — | — | 191.4 | — | — | — | 47.7 | 20.3 | 4.57 | 3.42 |
| Day 21 | 8.49 | 81 | 246 | 1.79 | — | 106.7 | 7.9 | 3.1 | — | 39.3 | 24.2 | 3.89 | 2.75 |
| Day 23 | 6.32 | 82 | 219 | 0.49 | — | 84.2 | 5.9 | 3.2 | — | 39.6 | 26 | 2.77 | 2.51 |
| Readmission Day 0 | 12.84 | 124 | 271 | 77.3 | 23 | 335.3 | 30.5 | 16.4 | 332 | 106.9 | 78.8 | 0.76 | 6.21 |
| Readmission Day 1 | 19.39 | 102 | 223 | 195.56 | 74.8 | 254 | 76.1 | 31 | 418 | 76.7 | 21.4 | 1.26 | 7.55 |
| Readmission Day 2 | 8.22 | 99 | 71 | 180.8 | 160.1 | 289.8 | 70.4 | 20.3 | 1235 | 70.3 | 23.9 | 3.44 | 7.35 |

**Notes:**WBC: white blood cell count; Hb: haemoglobin; PLT: platelet count; CRP: C-reactive protein; PCT: procalcitonin; Cr: serum creatinine; TB: total bilirubin; DB: direct bilirubin; LDH: lactate dehydrogenase; APTT: activated partial thromboplastin time; PT: prothrombin time; D-Dimer: D-dimer; FIB: fibrinogen;

Units and reference ranges: WBC ×10⁹/L (Ref: 3.5–9.5); Hb g/L (Ref: 115–150); PLT ×10⁹/L (Ref: 125–350); CRP mg/L (Ref: <5); PCT ng/mL (Ref: <0.05); Cr µmol/L (Ref: 45–90 in females); TB µmol/L (Ref: 5–21); DB µmol/L (Ref: 0–7); LDH U/L (Ref: 135–225); APTT s (Ref: 25–35); PT s (Ref: 11–15); D-Dimer mg/L FEU (Ref: <0.5);FIB g/L(Ref:2.0–4.0).“—” indicates that the parameter was not measured or the value was unavailable.
