# Supplementary material for: Case report of a fatal probable catastrophic antiphospholipid syndrome
Source: Front Med (Lausanne). 2026 Apr 14;13:1752865. doi: 10.3389/fmed.2026.1752865 (PMC13121309; doi:10.3389/fmed.2026.1752865)
Supplement: Supplementary file 3 [file Table_3.docx]

# Supplementary Table S3A. Timeline of Microbiological Investigations

This table summarizes all microbiological investigations performed during both hospitalizations. “–” indicates that the corresponding test was not performed on that day.

| Hospital Day | Bacterial Culture | Fungal Culture | Viral Nucleic Acid (PCR Panel) | NGS (Blood / BALF) | Serum G Test | Serum GM Test | Blood Culture |
| --- | --- | --- | --- | --- | --- | --- | --- |
| Day 0 | Sputum & stool: Negative | Negative | – | – | Negative | Negative | Negative |
| Day 1 | – | – | – | Blood NGS: Negative | – | – | – |
| Day 2 | – | – | – | – | – | – | Negative |
| Day 3 | – | – | – | – | – | – | Negative |
| Day 4 | Sputum & stool: Negative | – | – | BALF NGS: Negative | – | – | Negative |
| Day 5 | Stool: Negative | – | EBV, CMV, Mycoplasma, Influenza A/B, RSV, Parainfluenza, hMPV, Coronavirus, H3N2, H1N1, Chlamydia, Bocavirus: All Negative | – | – | – | Negative |
| Day 6 | – | – | – | – | – | – | Negative |
| Day 7 | – | – | – | – | – | – | – |
| Day 8 | BALF: Negative | BALF: Negative | – | – | – | – | – |
| Day 9 | – | – | – | – | – | – | – |
| Day 12 | – | – | – | – | – | – | Negative |
| Re-admission Day 0 | Sputum & stool: Negative | – | Same respiratory viral panel: All Negative | – | Negative | Negative | Negative |
| Re-admission Day 1 | – | – | – | – | – | – | Negative |
| Re-admission Day 2 | – | – | – | – | – | – | Negative |
| Re-admission Day 3 | – | – | – | – | – | – | – |

Abbreviations: BALF, bronchoalveolar lavage fluid; NGS, metagenomic next-generation sequencing; EBV, Epstein–Barr virus; CMV, cytomegalovirus; RSV, respiratory syncytial virus; hMPV, human metapneumovirus; GM, galactomannan.

## Notes: This table is provided to document the extent, timing, and breadth of pathogen investigations. Supplementary Table S3B. Arterial Blood Gas Analysis (All Values Converted to mmHg)

| Admission #1 – Hospital Day | PaO₂ (mmHg) | PaCO₂ (mmHg) | HCO₃⁻ (mmol/L) | BE (mmol/L) | Lactate (mmol/L) | FiO₂ (%) | P/F ratio |
| --- | --- | --- | --- | --- | --- | --- | --- |
| Day0 | 171.0 | 30.5 | 16.2 | -9.6 | 1.2 | 40 | 427.5 |
| Day1 | 171.8 | 33.0 | 20.0 | -4.5 | 0.7 | 40 | 429.4 |
| Day2 | 120.0 | 38.0 | 22.9 | -2.1 | 0.7 | 21 | 571.4 |
| Day3 | 112.5 | 36.2 | 21.8 | -3.1 | 0.7 | 21 | 535.7 |
| Day4 | 87.0 | 40.8 | 24.5 | -0.6 | 0.7 | 60 | 145.0 |
| Day5 | 182.3 | 36.8 | 24.0 | -0.4 | 0.7 | 50 | 364.5 |
| Day6 | 84.0 | 43.9 | 25.4 | 0.1 | 0.8 | 40 | 210.0 |
| Day7 | 115.5 | 32.3 | 22.5 | -1.5 | 1.7 | 40 | 288.8 |
| Day8 | 151.5 | 41.1 | 29.0 | 5.1 | 1.6 | 60 | 252.5 |
| Day9 | 66.5 | 43.7 | 34.2 | 11.1 | 1.5 | 40 | 166.1 |
| Day10 | 114.8 | 48.8 | 39.1 | 14.6 | 1.6 | NA | NA |
| Day11 | 190.5 | 38.1 | 28.8 | 5.4 | 3.2 | 21 | 907.5 |
| Day12 | 157.5 | 31.7 | 28.4 | 6.1 | 2.1 | 33 | 477.3 |
| Day13 | 177.8 | 34.7 | 24.0 | 0.0 | 1.3 | 33 | 538.2 |

Notes: Reference ranges — PaO₂: 80–100 mmHg; PaCO₂: 35–45 mmHg; HCO₃⁻: 22–26 mmol/L; Base excess (BE): −2 to +2 mmol/L; Lactate: 0.5–2.0 mmol/L. FiO₂ is expressed as percentage. P/F ratio was calculated as PaO₂/FiO₂. Reference ranges may vary slightly according to laboratory standards.

## Supplementary Table S3C. Norepinephrine Dosage During Hospitalization

| Hospital Day | Norepinephrine dose (μg/kg/min) |
| --- | --- |
| Day0 | 0.5 |
| Day1 | 0.5 |
| Day2 | 0.25–0.5 |
| Day3 | 0.25–0.5 |
| Day4 | 0.25–0.5 |
| Day5 | 0.25–0.5 |
| Day6 | 0.25–0.5 |
| Day7 | 0.25–0.5 |
| Day8 | 0.25–0.5 |
| Day9 | 0 |
| Day10 | 0 |
| Day11 | 0 |
| Day12 | 0 |
| Day13 | 0 |
| Day14 | 0 |
| Day15 | 0 |
| Day16 | 0 |
| Day17 | 0 |
| Day18 | 0 |
| Day19 | 0 |
| Day20 | 0 |
| Day21 | 0 |
| Day22 | 0 |
| Day23 | 0 |
| Day24 | 0 |
| Day25 | 0 |
| Readmission Day 0 | 0 |
| Readmission Day 1 | 0.5 |
| Readmission Day 2 | 0.5 |
| Readmission Day 3 | 0.5 |

Notes: Norepinephrine dosage is expressed as μg/kg/min. A value of 0 indicates discontinuation of vasopressor support. All blank entries in original records were recorded as 0 to indicate no norepinephrine administration.
