# Supplementary figures and images for: Case report of a fatal probable catastrophic antiphospholipid syndrome
Source: Front Med (Lausanne). 2026 Apr 14;13:1752865. doi: 10.3389/fmed.2026.1752865 (PMC13121309; doi:10.3389/fmed.2026.1752865)

**Supplementary Figure S4**
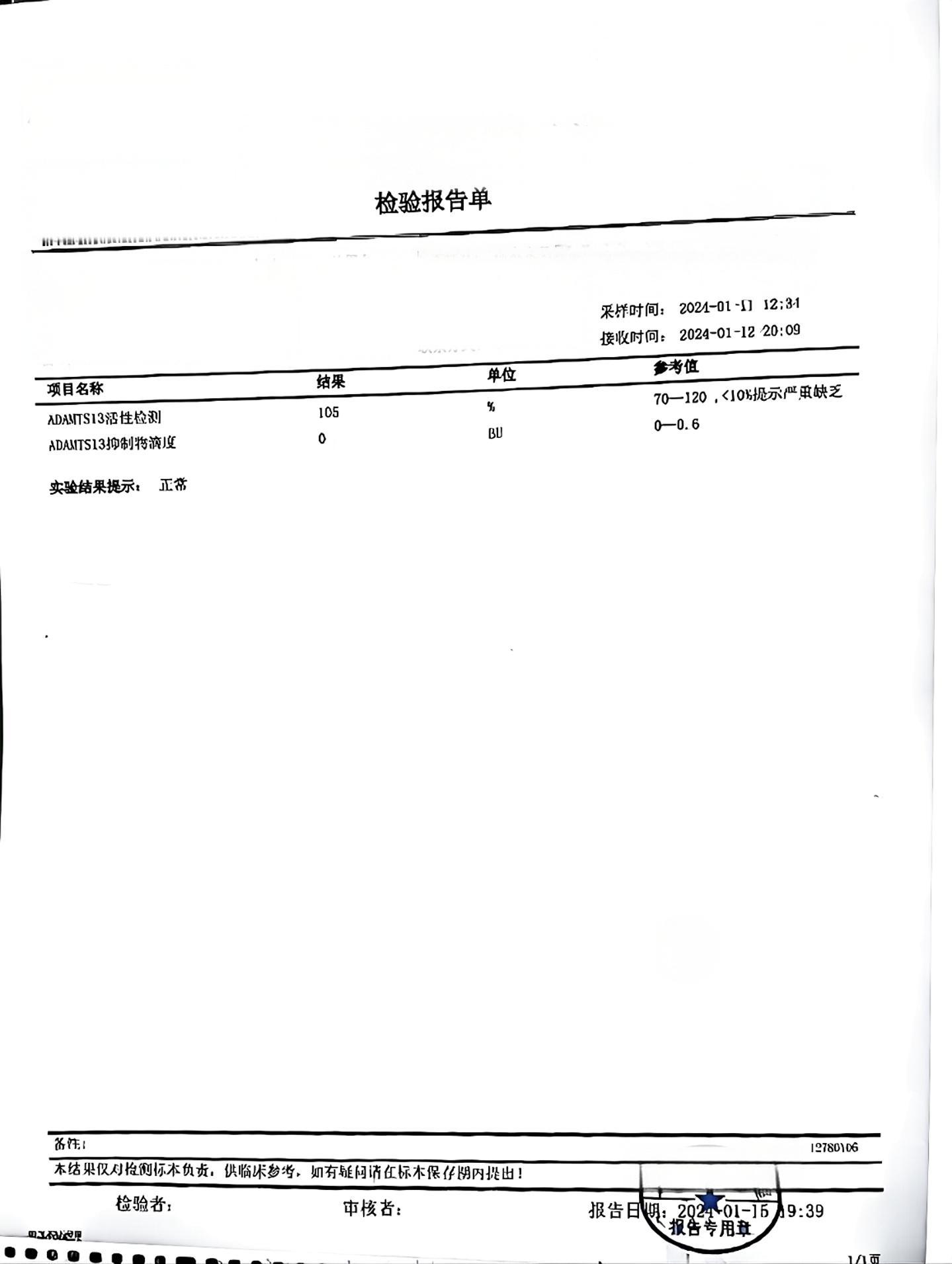

Supplement: Supplementary file 4 [file Table_4.docx]
