# Supplementary material for: Case report of a fatal probable catastrophic antiphospholipid syndrome
Source: Front Med (Lausanne). 2026 Apr 14;13:1752865. doi: 10.3389/fmed.2026.1752865 (PMC13121309; doi:10.3389/fmed.2026.1752865)
Supplement: Supplementary file 5 [file Table_5.docx]

**Supplementary Figure S5. Abdominal CT during the recurrent admission**


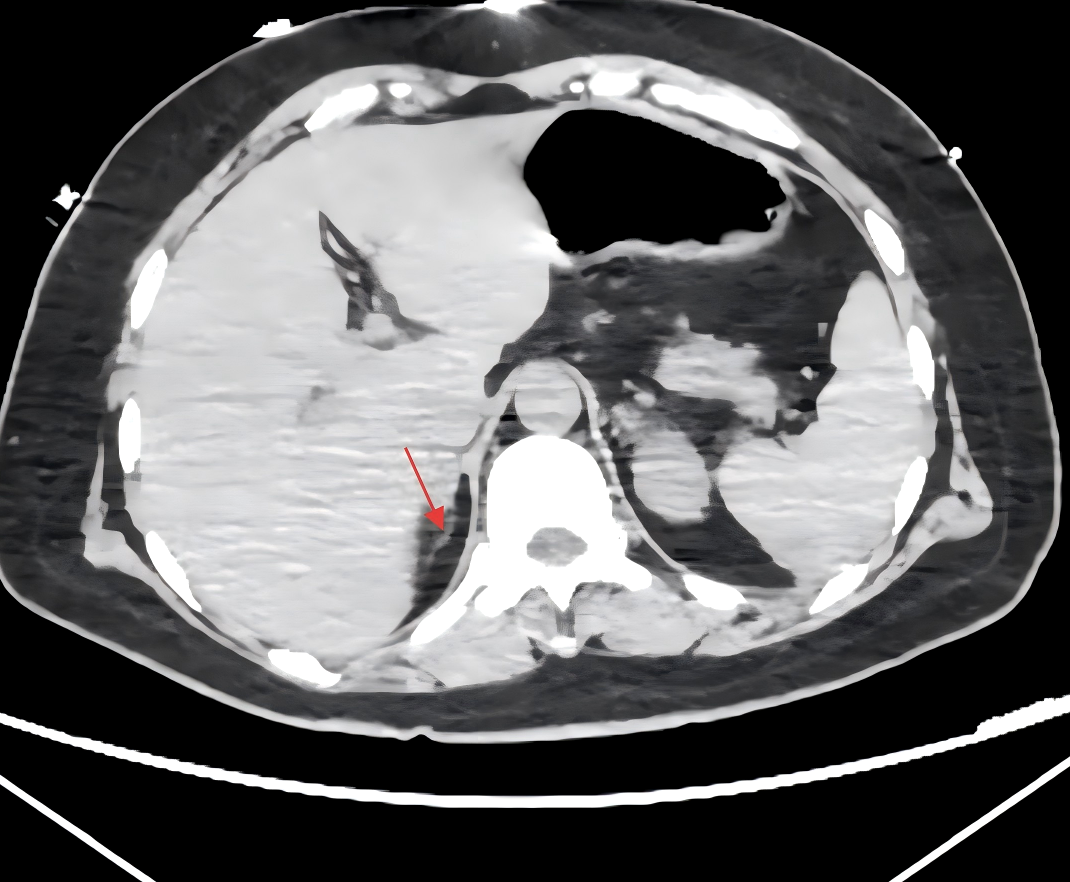


Notes:Red arrow indicates hyperdensity in the right adrenal gland.
